# Electrophysiological and behavioral indicators of musical knowledge about unfamiliar music

**DOI:** 10.1038/s41598-021-04211-w

**Published:** 2022-01-10

**Authors:** Anja-Xiaoxing Cui, Nikolaus F. Troje, Lola L. Cuddy

**Affiliations:** 1 Queen’s University, Kingston, Canada; 2 University of British Columbia, Vancouver, Canada; 3 York University, Toronto, Canada

**Keywords:** Cognitive neuroscience, Perception

## Abstract

Most listeners possess sophisticated knowledge about the music around them without being aware of it or its intricacies. Previous research shows that we develop such knowledge through exposure. This knowledge can then be assessed using behavioral and neurophysiological measures. It remains unknown however, which neurophysiological measures accompany the *development* of musical long-term knowledge. In this series of experiments, we first identified a potential ERP marker of musical long-term knowledge by comparing EEG activity following musically unexpected and expected tones within the context of known music (*n* = 30). We then validated the marker by showing that it does not differentiate between such tones within the context of unknown music (*n* = 34). In a third experiment, we exposed participants to unknown music (*n* = 40) and compared EEG data before and after exposure to explore effects of time. Although listeners’ behavior indicated musical long-term knowledge, we did not find any effects of time on the ERP marker. Instead, the relationship between behavioral and EEG data suggests musical long-term knowledge may have formed before we could confirm its presence through behavioral measures. Listeners are thus not only knowledgeable about music but seem to also be incredibly fast music learners.

## Introduction

Even non-musicians possess sophisticated knowledge about the music in their environment contrary to what many may think of themselves. For example, without possessing the technical vocabulary to describe the Neapolitan chord or its harmonic function, listeners’ brain activity differs when this chord is presented in a harmonically unexpected position rather than in an expected position^1^. Many studies converge on the finding that Western listeners possess implicit knowledge about the diatonic music system^2–5^. This diatonic music system uses a subset of seven tones of the chromatic scale, the twelve tones making up an octave, and forms the basis of most Western music.

Knowledge about the diatonic music system also includes the gradations in importance of and relationships between the various scale tones, which are summarized in the tonal hierarchy^6^. The tonal hierarchy reflects for example, that the fifth diatonic scale tone is more closely related to the first diatonic scale tone than other tones even when those may be closer to the first diatonic scale tone in frequency. Importantly, stimuli used to activate listeners’ representation about the diatonic music system do not need to mirror the intricacies of the tonal hierarchy. Even when the stimulus consists purely of a sequence in which each diatonic scale tone occurs the same number of times, listeners’ responses provide evidence of a more fine-grained representation of the tonal hierarchy. Similarly, when the stimulus provides only sparse information, e.g., it is a chord consisting of three of the seven diatonic scale notes, listeners’ responses imply that knowledge about the unheard notes’ relationship to the heard notes is present^7^.

Developmental^8,9^, cross-cultural^10,11^, and laboratory data^12,13^ show that musical knowledge is gained about the music in one’s environment. Other studies have extended this research by showing that this knowledge exerts influence on the perception of incoming tone sequences^14,15^. For example, when presented with intervals of two notes in the context of diatonic tone sequences, listeners’ perception of the distance between the two notes is influenced by the structural relationship between the two notes in diatonic music^16^.

Apart from behavioral indicators, researchers have also used neurophysiological markers to show the presence of long-term knowledge about musical syntax^17–20^ and common melodies^21,22^. Some have also used such markers to show the presence of knowledge about musical regularities underlying the stimuli presented within an experiment^23–29^. However, it is unknown what happens when the latter type of knowledge, i.e., knowledge developed within an experiment, *becomes* the former type of knowledge, i.e., knowledge that can be activated with a stimulus with sparse information. That is, we know little about the process of acquiring musical long-term knowledge itself.

Our goal in the present series of experiments was to study the neurophysiological correlates of developing musical long-term knowledge about hierarchies. Note that this knowledge is distinct from knowledge about musical syntax (see ref.^30^, p. 16), for which previous research has identified the early right anterior negativity^18^ as a neurophysiological marker. In fact, recent results^31^ show that the early right anterior negativity does not map clearly onto knowledge about tonal hierarchies.

To meet our goal, we proceeded in three steps: First, we explored what EEG activity differentiates between incongruent and congruent tones in a music system, for which Western listeners are known to possess implicit long-term knowledge^7^. In the next experiment, we tested whether this EEG activity is a specific indicator of congruency such that it does not indicate congruency when participants do not possess long-term knowledge. Lastly, we tested whether this EEG activity emerges with the development of musical long-term knowledge about a previously unknown music system.

## Results

### Experiment 1

As outlined in the introduction, Western listeners possess implicit knowledge about the diatonic music system. As such, we can utilize its properties to explore what EEG activity differentiates between diatonic and non-diatonic probe tones, i.e., musically expected and unexpected tones within the context of a music system of which Western listeners possess long-term knowledge. With the presentation of a diatonic tone sequence, Western listeners’ representation of diatonic music is assumed to be activated. When a non-diatonic probe tone then occurs, their expectations are violated, potentially resulting in different EEG activity compared to when a diatonic probe tone is presented.

In Experiment 1, participants listened to diatonic tone sequences followed by diatonic or non-diatonic probe tones. We asked participants to indicate each probe tone’s fit to the tone sequences using a two-alternative forced choice while we collected their EEG data. Differences in mean amplitude in a visually identified time window were analyzed statistically to determine whether brain activity differentiated between diatonic and non-diatonic probe tones, which can be considered as congruent and incongruent respectively.

**Figure 1:**
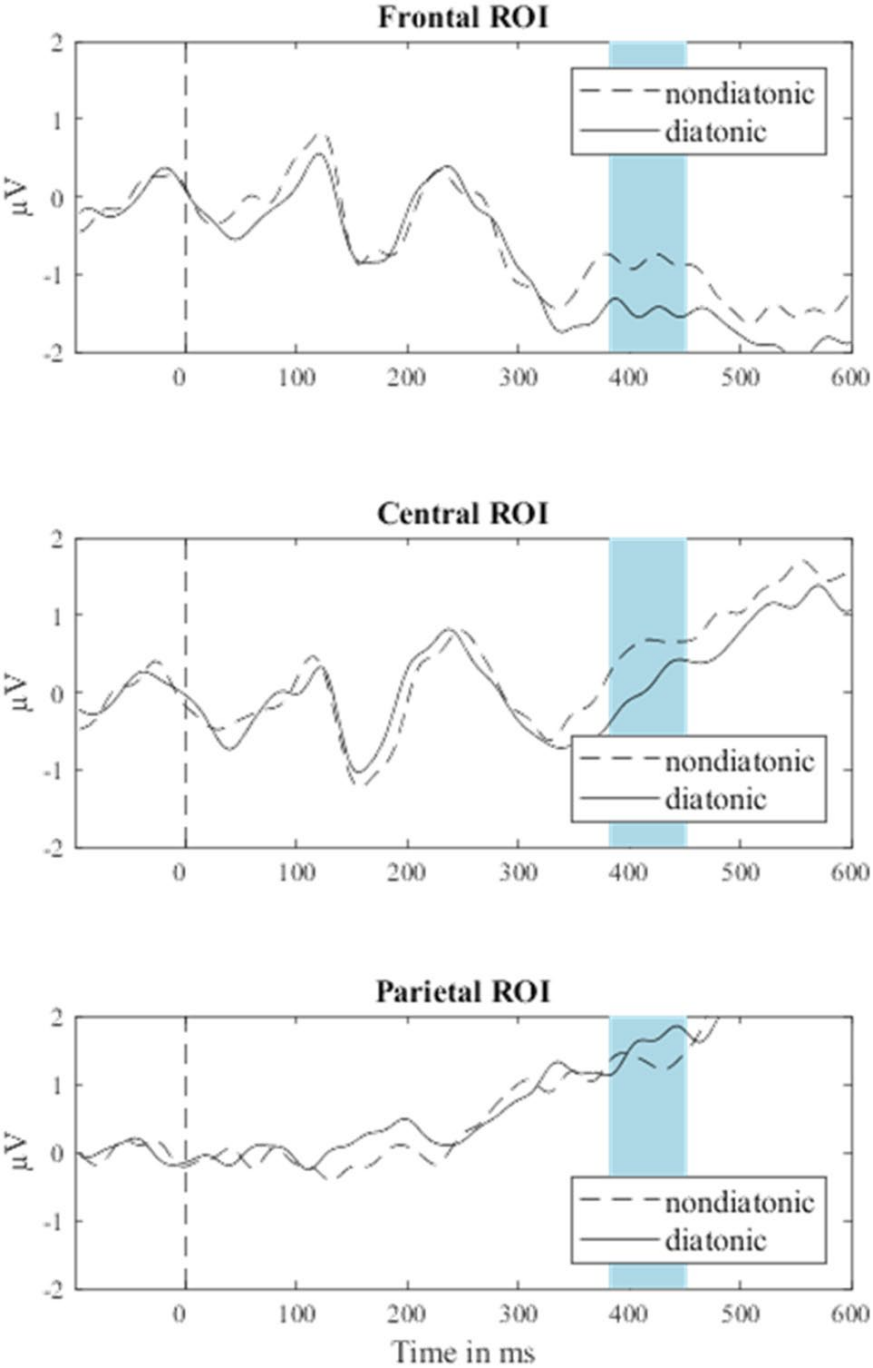
Average EEG activity at three ROIs following non-diatonic, i.e., incongruent, and diatonic, i.e., congruent probe tones. The time window of interest is shaded in blue. ROIs were formed by averaging signals across electrodes around three midline electrodes, Fz, Cz, and Pz, based on the morphology of the EEG net (channel numbers indicate the electrode nomenclature of the Geodesic Sensor Net—frontal: 4, 5, 11 (Fz), 12, 16, 19; central: 7, 31, 55, 80 106, 129 (Cz); parietal: 61, 62 (Pz), 67, 72, 77, 78).

An ANOVA with factors ROI (frontal, central, parietal) and Congruency (congruent: C, G, incongruent: C♯, F♯) was calculated on the mean amplitudes in the time window of 380 ms to 450 ms. This analysis yielded a significant main effect of ROI, *F*(2,58) = 29.97, *p* < 0.001, η^2^ = 0.508. There was no significant main effect of Congruency, *F*(2,58) = 3.35, *p* = . 078, η^2^ = 0.104. However, the interaction of ROI and Congruency was significant, *F*(2,58) = 4.21, *p* = . 020, η^2^ = 0.127. Average activity at all three ROIs is shown in Fig. 1. Follow-up *t*-tests showed that the difference between congruent and incongruent trials was significant for the frontal and central ROI, *t*frontal(29) = 2.21, *p* = 0.035, *d* = 0.404, *t*central(29) = 2.46, *p* = 0.020, *d* = 0.449, with incongruent trials eliciting more positive activity than congruent trials. Signals obtained from the parietal ROI were not significantly different between incongruent and congruent trials, *t*parietal(29) = 1.12, *p* = 0.271, *d* = 0.205.

To analyze the behavioral data, we calculated a probe-tone rating for each level of Congruency from the proportion of times that participants indicated that the probe-tone fit rather than did not fit on a two-alternative forced choice. Paired samples *t*-test showed that probe-tone ratings were higher for congruent tones (*M* = 87%, *SD* = 13%) than for incongruent tones (*M* = 8%, *SD* = 11%), *t*(29) = 20.27, *p* < 0.001, *d* = 3.701. Repeated measures correlation between behavioral and electrophysiological responses was significant at the frontal ROI, *r*(89) = -0.31, *p* = 0.003, and central ROI, *r*(89) = -0.40, *p* < 0.001, but not at the parietal ROI, *r*(89) = 0.17, *p* = 0.107.

This experiment was conducted to identify a potential ERP marker for musical long-term knowledge about tonal hierarchies. Participants overwhelmingly rated diatonic tones as more fitting with diatonic tone sequences as expected. Their EEG recordings indicated a time-window between 380 and 450 ms in which EEG activity was significantly different at frontal and central electrodes between incongruent and congruent tones. We attempt a contextualization of this activity with previous research in the discussion. With the ensuing experiment, we wanted to validate this marker on a separate sample of participants as well as show that this marker does *not* differentiate between incongruent and congruent tones within an unfamiliar, non-diatonic music system.

### Experiment 2

We hypothesized that we would then see significant effects of congruency on mean amplitudes of the ERP marker identified in Experiment 1 in a block of trials with diatonic tone sequences, i.e., in a replication of Experiment 1. We further hypothesized that there would be no such effects in a block of trials with non-diatonic tone sequences. The central assumption is that Western listeners possess musical long-term knowledge about the former but not about the latter if the latter is carefully constructed to not correspond to diatonic music. The stimuli thus differ in their familiarity.

**Figure 2:**
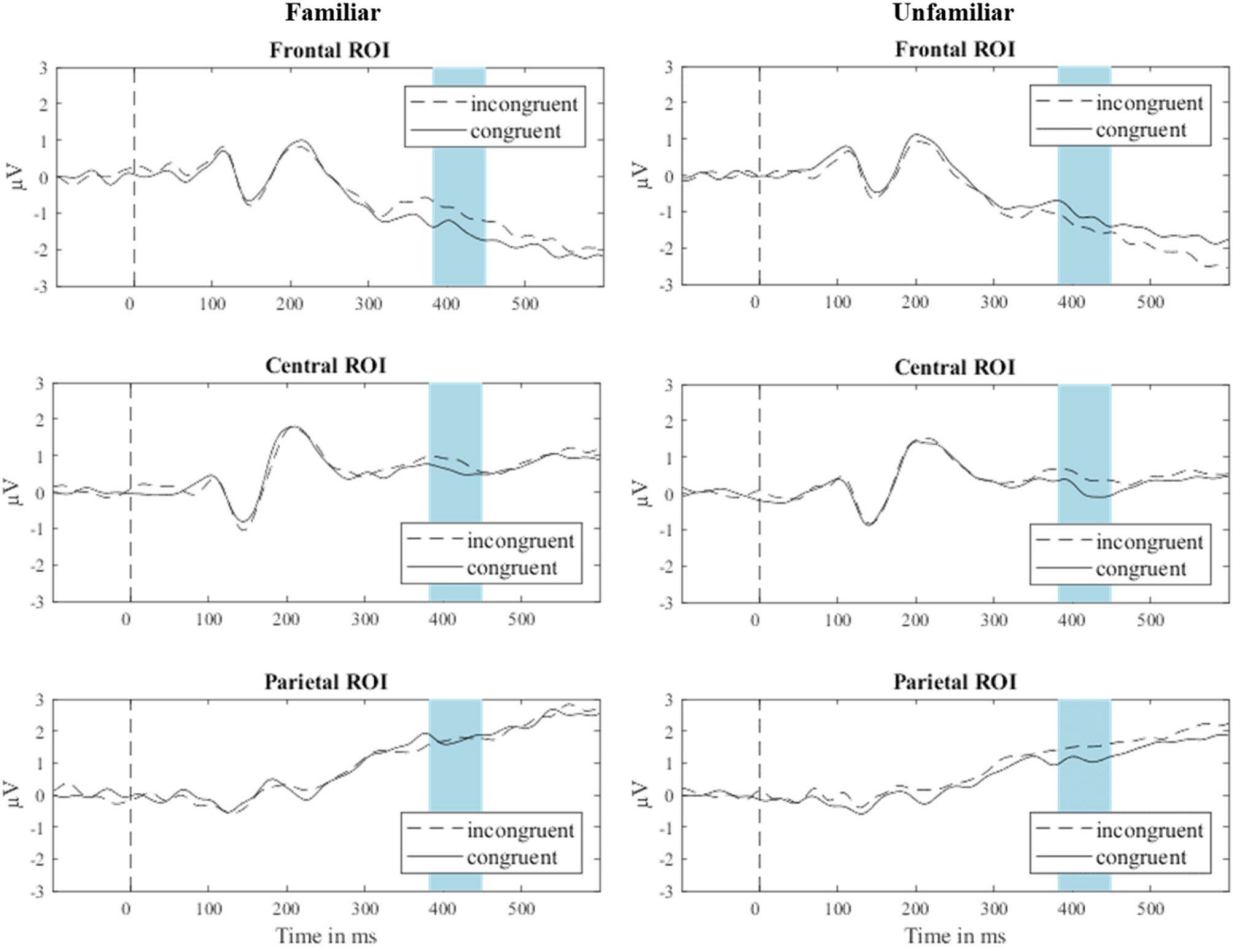
Average EEG activity at three ROIs following incongruent and congruent probe tones following tone sequences from the familiar and unfamiliar music systems. The time window of interest is shaded in blue. ROIs were formed by averaging signals across electrodes around three midline electrodes, Fz, Cz, and Pz, based on the morphology of the EEG net (channel numbers indicate the electrode nomenclature of the Geodesic Sensor Net—frontal: 4, 5, 11 (Fz), 12, 16, 19; central: 7, 31, 55, 80 106, 129 (Cz); parietal: 61, 62 (Pz), 67, 72, 77, 78).

Here, participants listened to diatonic tone sequences followed by diatonic, i.e., congruent, or non-diatonic, i.e., incongruent probe tones as well as tone sequences from an unfamiliar, non-diatonic music system followed by congruent and incongruent probe tones. They assessed each probe tone’s fit to the tone sequences while we collected their EEG data. Activity at the three midline ROIs is shown in Fig. 2. Differences in mean amplitude in the same time window used in Experiment 2 were analyzed statistically to determine whether brain activity differentiated between congruent and incongruent probe tones. While the tone sequences were generated from different music systems in the two blocks, the same probe tones were used in both blocks.

The mean amplitudes of the ERP marker identified in Experiment 1 at the three midline ROIs were analyzed in an ANOVA with factors Music System (familiar, unfamiliar), ROI (frontal, central, parietal), and Probe Tone (C, C♯, F♯, G). There were significant main effects of Music System, *F*(1,33) = 5.449, *p* = 0.026, η^2^ = 0.142, and ROI, *F*(2,66) = 48.153, *p* < 0.001, η^2^ = 0.593, but not Probe Tone, *F*(3,99) = 8.90, *p* = 0.449, η^2^ = 0.026. A significant three-way interaction of these factors was found, *F*(6,198) = 2.84, *p* = 0.011, η^2^ = 0.079. No other interaction effects were significant, *p*s > 0.05.

**Figure 3:**
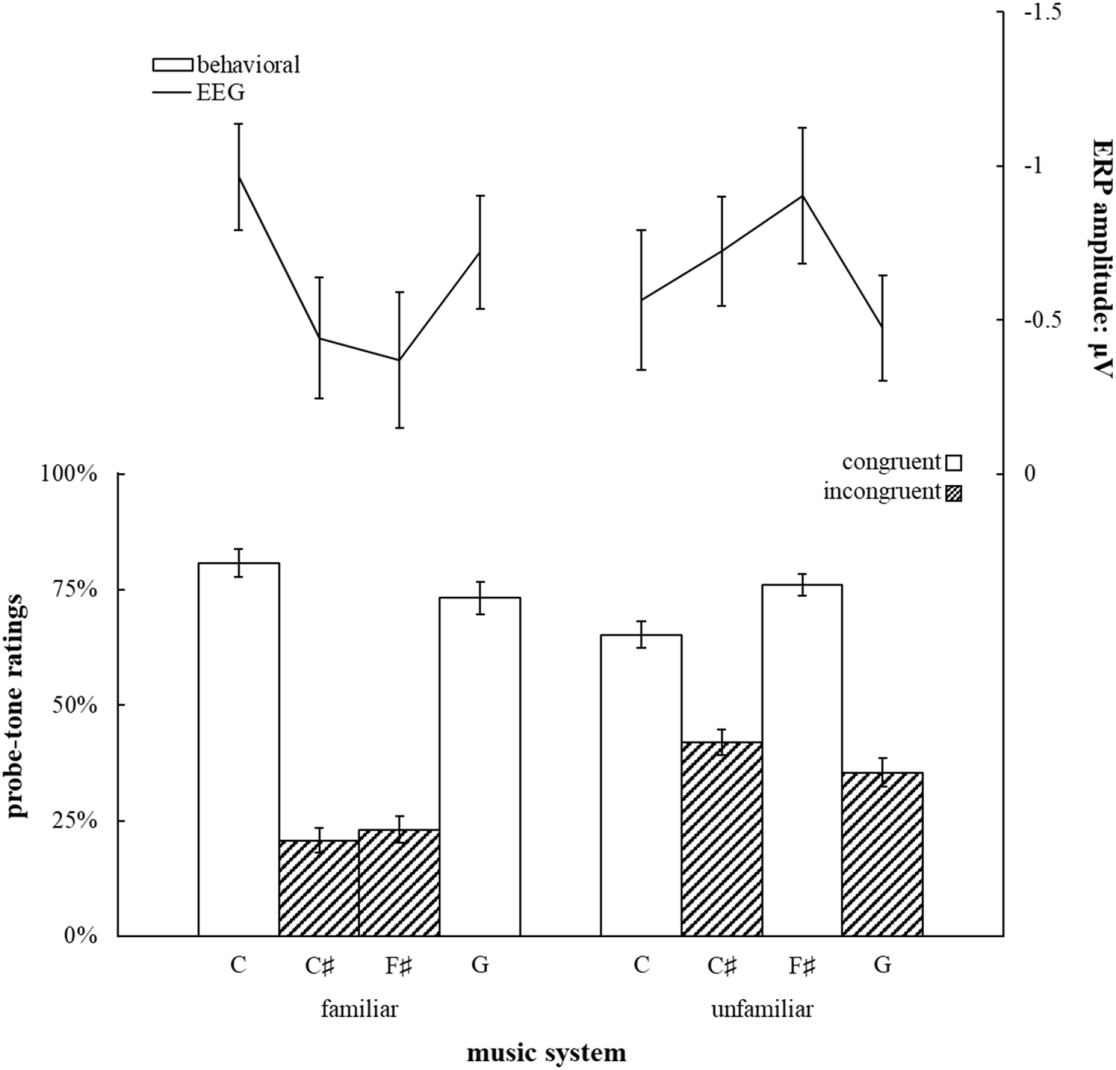
Average probe-tone ratings calculated from two-alternative forced choice probe-tone judgements in Experiment 2 for each probe tone with corresponding ERP amplitude at frontal electrodes over 40 ms centered around the positive peak in the time window identified in Experiment 1. Error bars indicate standard errors of the mean adjusted for between-subject variability. Patterned bars indicate averaged probe-tone ratings for incongruent probe tones. Empty bars indicate averaged probe-tone ratings for congruent probe tones.

To investigate what drove the significant three-way interaction, follow-up ANOVAs with the factors Congruency (congruent_familiar_: C, G, incongruent_familiar_: C♯, F♯; congruent_unfamiliar_: C, F♯, incongruent_unfamiliar_: C♯, G) and Tone Height (low: C, C♯, high: F♯, G) were conducted for each music system and for each ROI separately. There were no significant main effects of Tone Height nor significant interactions of Congruency and Tone Height at any ROI, *p*s > 0.05. The main effect of Congruency was significant at frontal electrodes for the familiar music system, *F*(1,33) = 8.83, *p* = 0.006, η^2^ = 0.211, but not for the unfamiliar system, *F*(1,33) = 0.52, *p* = 0.476, η^2^ = 0.015, nor at any other ROI, *p*s > 0.05. Mean amplitudes at frontal electrodes for each probe tone are shown in Fig. 3 along average probe-tone ratings.

The analysis of our behavioral data showed significant main effects of Music System, *F*(1,33) = 13.25, *p* = 0.001, η^2^ = 0.287, and Probe Tone, *F*(1,33) = 46.04, *p* < 0.001, η^2^ = 0.582, as well as a significant interaction between Music System and Probe Tone on probe-tone ratings, *F*(3,99) = 94.94, *p* < 0.001, η^2^ = 0.742. Similar to our follow-ups on the electrophysiological data, ANOVAs with factors Congruency (congruent_familiar_: C, G, incongruent_familiar_: C♯, F♯; congruent_unfamiliar_: C, F♯, incongruent_unfamiliar_: C♯, G) and Tone Height (low: C, C♯, high: F♯, G) were conducted to assess whether participants distinguished between congruent and incongruent probe tones for each music system separately. There was no significant main effect of Tone Height for either music system, *p*s > 0.05. There was a significant interaction of Congruency and Tone Height for both the familiar music system, *F*(1,33) = 7.04, *p* = 0.012, η^2^ = 0.176, and the unfamiliar system, *F*(1,33) = 15.77, *p* < 0.001, η^2^ = 0.322. However, the main effect of Congruency held over both levels of Tone Height for the familiar music system, *F*(1,33) = 156.15, *p* < 0.001, η^2^ = 0.826, as well as for the unfamiliar music system, *F*(1,33) = 116.43, *p* < 0.001, η^2^ = 0.779, such that congruent probe tones were judged fitting more often for both music systems.

Repeated measures correlation between behavioral and electrophysiological responses at the frontal ROI was significant for the familiar music system, *r*(101) = -0.26, *p* = 0.009, but not for the unfamiliar music system, *r*(101) = -0.08, *p* = 0.424.

Thus, the ERP marker identified in Experiment 1 was again found to be different between incongruent and congruent tones in our participants when they were presented with diatonic tone sequences. Crucially, the ERP marker was *not* different between incongruent and congruent tones when our participants were presented with non-diatonic tone sequences. Given our assumption that Western listeners possess implicit long-term knowledge of diatonic music, we can thus assume that the ERP marker is specifically a marker for musical long-term knowledge and not a marker of musical knowledge that may be gained within the time frame of the experiment. In Experiment 3 we set out to study whether newly developed musical long-term knowledge about a previously unknown music system may also be indicated by this marker.

### Experiment 3

We hypothesized that behavioral responses indicating the presence of newly formed musical long-term knowledge should be accompanied by the emergence of the ERP marker identified in Experiment 1 and validated in Experiment 2. To test this, we utilized a behavioral paradigm described in previous research^32^. Using this paradigm, the presence of musical long-term knowledge about a previously unknown music system can be explored.

In the paradigm, probe-tone judgements about an unfamiliar music system are made in two blocks separated by an exposure phase. During the latter, new information about the unfamiliar music system is introduced, meaning that some tones occur only during the exposure phase. These tones are not part of the probe-tone contexts, the stimuli used to elicit probe-tone judgments. The probe-tone contexts thus contain only sparse information and specific comparisons can then test whether listeners incorporated the information that was introduced during the exposure phase into their representation of the music system by comparing their probe-tone judgments before and after the exposure phase.

Responses to four probe-tone categories are compared that are defined by whether or not the probe tones occur during the different phases of the experiment. Some probe tones may be part of probe-tone contexts used to elicit the probe-tone judgements and also be heard during the exposure phase. These tones are considered congruent throughout the experiment *C*. On the other hand, some probe tones may neither occur in probe-tone contexts nor during the exposure phase and are thus incongruent throughout the experiment *C*~. Then there are probe tones that are only congruent with respect to the probe-tone judgment blocks *C*_P_, which do not occur during the exposure phase, and probe tones that are only congruent with respect to the exposure phase *C*_E_ and are not part of the probe-tone contexts.

**Figure 4:**
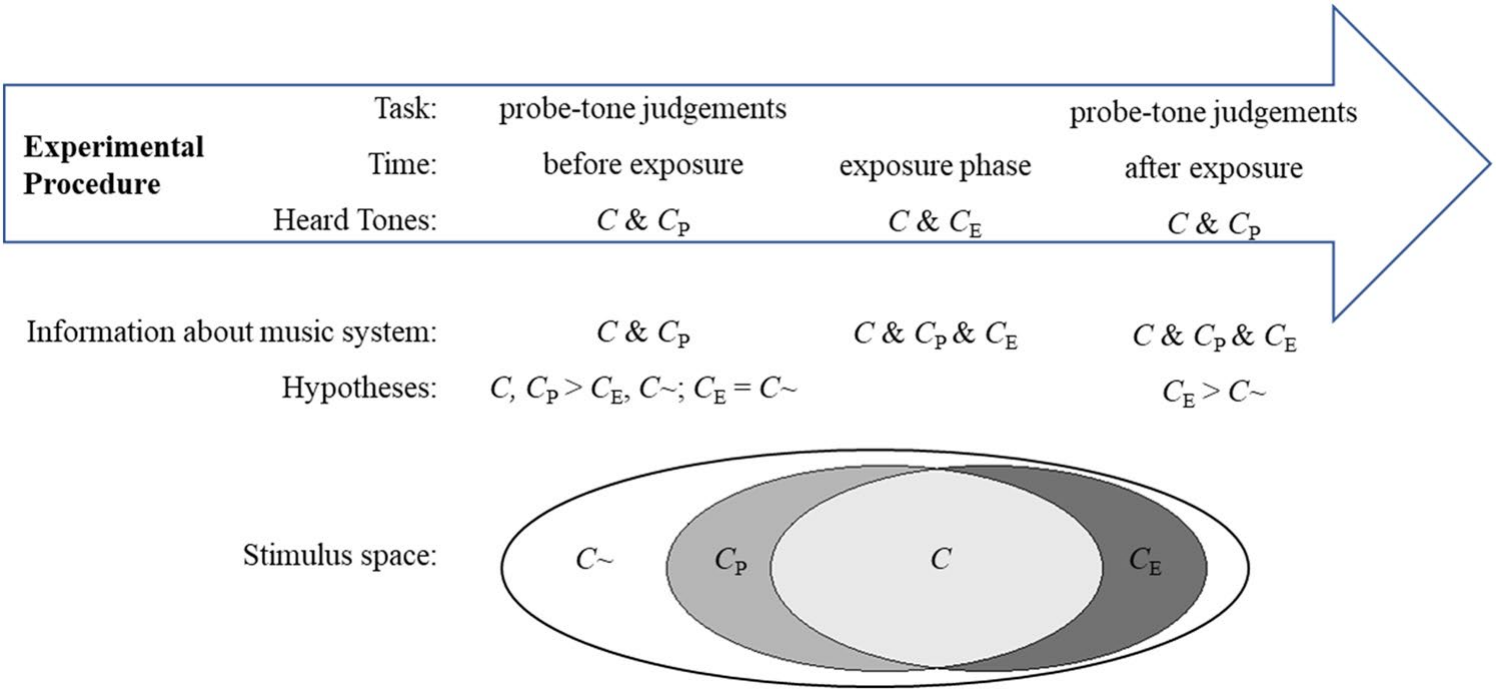
Schema of experimental procedure and hypotheses for Experiment 3. Participants give probe-tone judgements before and after an exposure phase. These judgements are elicited using probe-tone contexts using tones of two probe-tone categories (*C* and *C*_P_). During the exposure phase, participants hear tone sequences containing tones of probe-tone categories *C* and *C*_E_. Thus, new information (*C*_E_) about the music system is introduced during the exposure phase. Should this new information be incorporated into listeners’ representation of the music system, ratings for probe-tone category *C*_E_ are expected to be higher than ratings for probe-tone category *C*~ after the exposure phase. The stimulus space visualizes the four probe-tone categories as a Venn diagram: The music system comprises the grey components, while the white space contains tones that do not belong to the music system (*C*~).

Before the exposure phase, probe tones in categories *C* and *C*_P_, should thus receive higher ratings than probe tones in categories *C*_E_, and *C*~. However, while *C*_E_ tones do not occur in the tone sequences of the probe-tone judgment blocks, they are considered part of the unfamiliar music system and thus should receive higher ratings after they are “introduced” to the system during the exposure phase. At this point, only if their ratings increase can we say that participants gained musical long-term knowledge. The increased ratings would indicate that tones that are not immediately present in the auditory environment are also regarded as part of a music system. The paradigm and hypotheses are schematized in Fig. 4.

**Figure 5:**
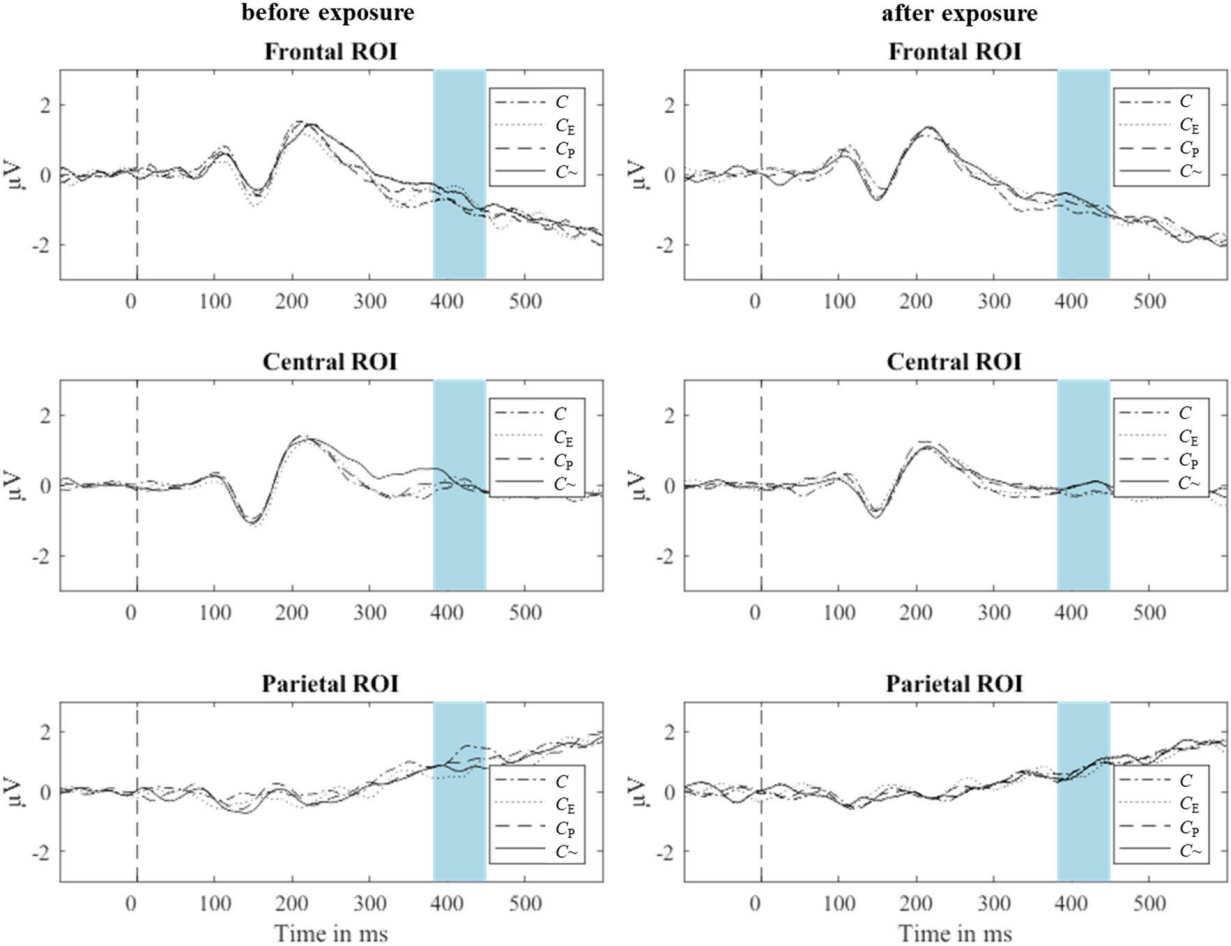
Average EEG activity at three ROIs following probe tones of four different probe-tone categories. The time window of interest is shaded in blue. ROIs were formed by averaging signals across electrodes around three midline electrodes, Fz, Cz, and Pz, based on the morphology of the EEG net (channel numbers indicate the electrode nomenclature of the Geodesic Sensor Net—frontal: 4, 5, 11 (Fz), 12, 16, 19; central: 7, 31, 55, 80 106, 129 (Cz); parietal: 61, 62 (Pz), 67, 72, 77, 78).

**Figure 6:**
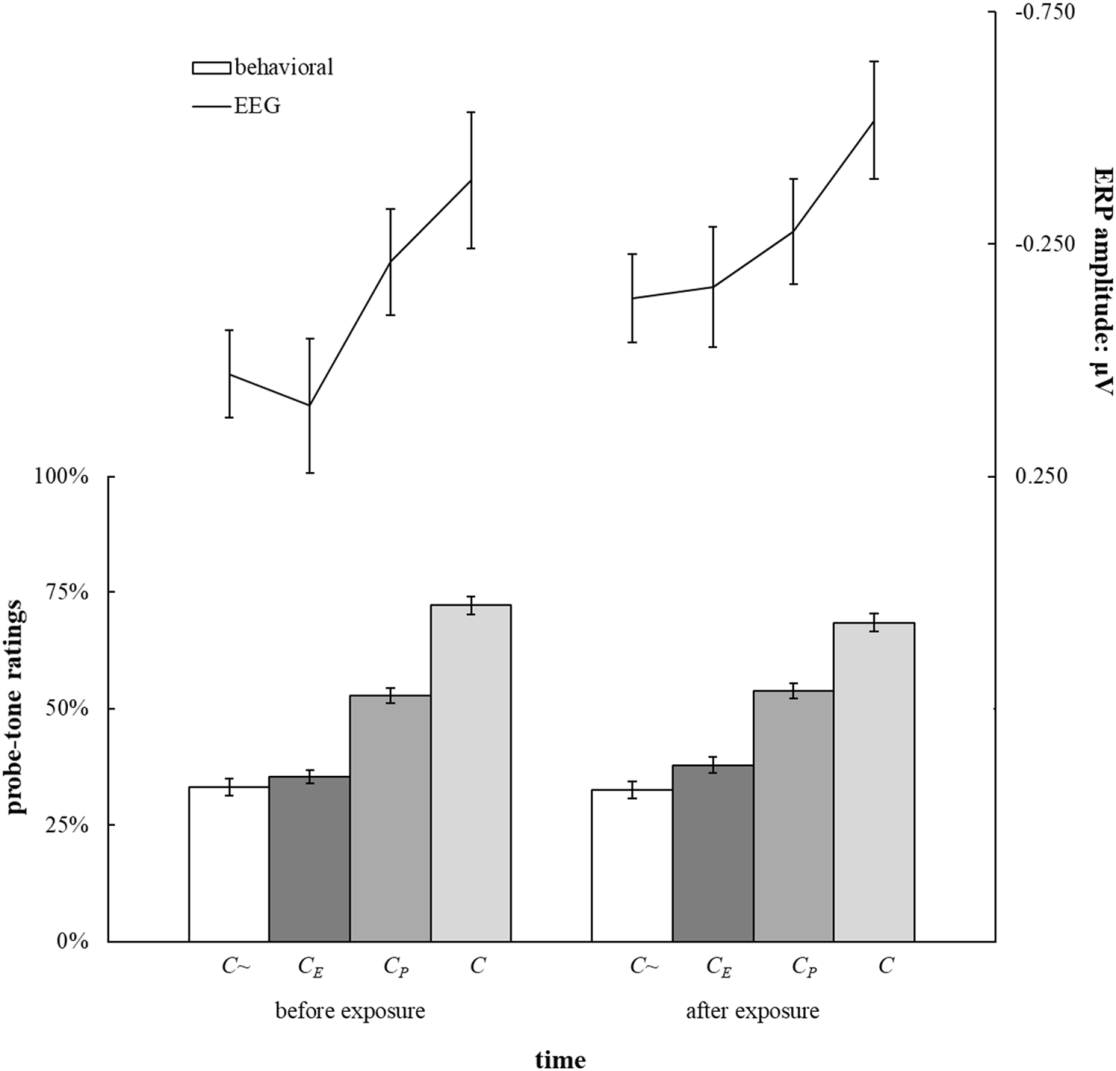
Average probe-tone ratings calculated from two-alternative forced choice probe-tone judgements in Experiment 3 for each probe tone with corresponding ERP amplitude at frontal electrodes over 40 ms centered around the positive peak in the time window identified in Experiment 1. Error bars indicate standard errors of the mean adjusted for between-subject variability. Colored bars indicate averaged probe-tone ratings for each probe-tone category. Note that roughly twice the number of trials is averaged for each datapoint here compared to Fig. 3, thus the smaller error bars.

We collected participants’ EEG data while they completed this paradigm. EEG activity at the midline electrodes is shown in Fig. 5. Figure 6 shows the mean amplitudes at the frontal ROI for each of the four categories of probe tones, *C*, *C*_P_, *C*_E_, and *C*~, before and after the exposure phase, as a line graph. Mean amplitudes for probe-tone category *C* are lowest as expected at frontal electrodes both before and after exposure. However, as outlined above, we specifically hypothesized that additionally, mean amplitudes for *C*~ would be greater than mean amplitudes for *C*_E_ but only after the exposure phase and at frontal or central electrodes, which would result in a four-way interaction of Time (before or after the exposure phase), ROI (frontal, central, parietal), Exposure (*C* and *C*_E_ versus *C*_P_,and *C*~), and Probe-Tone Block (*C* and *C*_P_ versus *C*_E_, and *C*~).

This four-way interaction was not significant, *F*(2,36) = 0.11, *p* = 0.893, η^2^ = 0.006. The main effect of ROI was significant, *F*(2,36) = 23.65, *p* < 0.001, η^2^ = 0.568; all remaining main effects were not significant, *p*s > 0.05. The three-way interaction between Time, ROI, and Probe-Tone Block was not significant either, *F*(2,36) = 3.07, *p* = 0.059, η^2^ = 0.146, nor the three-way interaction between Time, ROI, and Exposure, *F*(2,36) = 0.31, *p* = 0.734, η^2^ = 0.017. However, there were significant two-way interactions between ROI and Exposure, *F*(2,36) = 4.61, *p* = 0.016, η^2^ = 0.204, and between ROI and Probe-Tone Block, *F*(2,36) = 4.39, *p* = 0.020, η^2^ = 0.196, such that amplitudes for tones incongruent with Exposure and Probe-Tone Block respectively were higher at frontal and central electrodes but lower at parietal electrodes. All remaining interaction effects were not significant, *p*s > 0.05.

Behavioral responses are shown in Fig. 6 as a bar graph and were analyzed using a mixed-effects model of all probe-tone ratings which was able to explain *R*^2^ = 0.78 of the variability. Several predictors were significant: Exposure, *t*(312) = 12.33, *p* < 0.001, OR = 1.26, Probe-Tone Block, *t*(312) = 31.01, *p* < 0.001, OR = 1.81, and the interaction of Exposure and Probe-Tone Block, *t*(312) = 7.79, *p* < 0.001, OR = 1.16. Additionally, the three-way interaction of Time, Exposure, and Probe-Tone Block was a significant predictor, *t*(312) = 2.42, *p* = 0.016, OR = 1.05. All other predictors were not significant, *p*s > 0.05.

Behavioral responses obtained before exposure were entered into a second mixed-effects model. The predictor Probe-Tone Block was significant, *t*(156) = 22.78, *p* < 0.001, OR = 1.85, such that tones occurring in the probe-tone context yielded higher probe-tone ratings as expected. The predictor Exposure was significant, *t*(156) = 9.01, *p* < 0.001, OR = 1.28, as well as the interaction of Probe-Tone Block and Exposure, *t*(156) = 7.13, *p* < 0.001, OR = 1.21.

Contrast analyses revealed that the interaction between Probe-Tone Block and Exposure and the main effect of Exposure was driven by a significant difference between ratings for probe-tone category *C* and ratings for all other probe tone categories, *C*_P_: *t*(156) = 4.04, *p* < 0.001, OR = 1.20, *C*_E_: *t*(156) = 12.21, *p* < 0.001, OR = 1.76, *C*~: *t*(156) = 14.23, *p* < 0.001, OR = 1.95. The difference between ratings for probe-tone categories *C*~ shown as white bars in Fig. 4 and *C*_E_ shown in dark grey bars in Fig. 3 was not significant before exposure, *t* (39) = 1.17, *p* = 0.250, *d* = 0.185, but was significant after exposure, *t*(39) = 2.39, *p* = 0.022, *d* = 0.378, such that ratings for probe-tone category *C*_E_ were higher than ratings for probe-tone category *C*~.

There was a significant correlation between behavioral and electrophysiological responses at the frontal ROI before exposure, *r*(265) = −0.15, *p* = 0.013. This correlation was not significant after exposure, *r*(265) = −0.10, *p* = 0.100.

## Discussion

In Experiment 1, we searched for a potential ERP marker for musical long-term knowledge about tonal hierarchies. Participants’ EEG recordings indicated a time-window between 380 and 450 ms in which EEG activity significantly differed between incongruent and congruent tones. We confirmed that this was a marker of musical long-term knowledge by replicating its occurrence in Experiment 2 using stimuli, of which participants are presumed to possess musical long-term knowledge, and by demonstrating its absence using stimuli, of which participants cannot possess musical long-term knowledge. We next set out to track this ERP marker in another experiment, in which participants develop musical long-term knowledge after an exposure phase.

Contrary to our expectations, the ERP marker was not different after exposure in Experiment 3. This finding is particularly surprising, given that our behavioral data indicate that participants possessed musical knowledge about probe tones which did not immediately occur in the auditory environment. There are two possibilities for this finding: Either, we did not identify the appropriate ERP marker for this type of knowledge in Experiments 1 and 2, or the musical long-term knowledge on which we benchmarked the ERP marker in Experiments 1 and 2 is not the same type of knowledge as that shown by our participants after exposure in Experiment 3. We next consider both possibilities and contextualize the EEG activity which we used as our marker with prior research on neurophysiological correlates of musical knowledge.

Regarding the first possibility, we have argued that the findings from Experiments 1 and 2 identify a component that may serve specifically as an indicator of musical long-term knowledge. In Experiment 1, we identified a time window during which activity at frontal and central electrodes differentiated non-diatonic from diatonic tones. Specifically, activity at frontal and central electrodes was more positive for non-diatonic probe tones than for diatonic probe tones in a time window of 380 ms to 450 ms. This late positivity is akin to that commonly described as the P3b component which is shown to be more positive for musically unexpected events^17,33–36^.

Other researchers have also identified this late time window in which changes or differences may indicate explicitly learned structure in auditory stimuli when the structure is based on the frequency of occurrence of auditory events^37,38^. These events can be a sequence of two tones or^37^ or speech sounds whose voice onset time is modulated^38^. Our findings thus relate to these earlier findings on auditory statistical learning in that the learned structure in our stimuli is based on the frequency of occurrence of multiple different scale tones.

Differences found in earlier time windows, e.g., the mismatch negativity or the early right anterior negativity, are ascribed to inattentive processes that take place when the listener is not explicitly attending to the stimuli^31,37^. These ERPs reflect different neurocognitive functions: Specifically, the mismatch negativity cooccurs with unexpected events based on auditory memory and the early right anterior negativity cooccurs with unexpected events based on music-syntactical expectations^39^. However, in contrast to the ERP marker found in our study, these earlier neurophysiological differences are found to not systematically relate to behavioral ratings: For example, a recent paper showed that the relationship between the early right anterior negativity and probe-tone ratings differed greatly between participants^31^. Note however that the authors of the paper employed a passive task, in which participants did not attend to the stimuli while their neurophysiological data were collected. In contrast, we employed an active task, as did other authors who showed a consistent relationship between behavioral and later ERP measures^37^.

Researchers posit the involvement of memory in forming the P3b component such that it is the comparison with memory that engages mechanisms associated with its production^40^. Given that non-diatonic probe tones can be regarded as a musically unexpected event, it would make sense that a P3b-like component also differentiates between non-diatonic and diatonic tones, i.e., tones external and internal to the diatonic music system. Results from Experiment 2 support this conclusion. Specifically, when listeners did not possess musical long-term knowledge about a music system, this activity did not differentiate between system-external and system-internal tones. Thus, we find it likely that the marker is indeed tracking musical long-term memory.

The second possibility mentioned above may be advanced as a more likely explanation for the absence of effects on the ERP marker in Experiment 3, i.e., that the knowledge indicated by the presence of the P3b-like component in Experiment 1 is not the same type of knowledge shown by listeners in Experiment 3 after the exposure phase. To this end, it is interesting to consider the results from the correlational analyses of behavioral and electrophysiological data from Experiments 2 and 3. While they were correlated in the block of trials in Experiment 2, in which listeners heard diatonic tone sequences, they did not correlate in the block of trials in Experiment 2, in which listeners heard tone sequences generated from an unfamiliar music system. Thus, the correlation was present in the block in which we assume musical long-term knowledge.

Behavioral and electrophysiological data also did not correlate in the block of probe-tone judgments after the exposure phase in Experiment 3. However, behavioral and electrophysiological data were correlated in the block of probe-tone judgments *before* the exposure phase in Experiment 3. This raises the possibility of some sort of musical long-term knowledge prior to the exposure phase already, specifically, about the relationship between *C* and *C*_P_ tones as tones occurring in the tone sequences to *C*~ and *C*_E_ as tones not occurring in the tone sequences.

Though the interaction between Time, ROI, and Probe-Tone Block was not significant, the P3b-like component was indeed more positive for *C*~ and *C*_E_ compared to *C* and *C*_P_ tones at frontal electrodes according to a post-hoc analysis of the mean amplitudes collected before the exposure phase, *F*(1,37) = 7.39, *p* = 0.010. This effect is also visible in Fig. 4. Given the doubled number of trials in the first block of trials in Experiment 3 compared to a single block of trials in Experiment 2 (160 trials compared to 80 trials), we cannot exclude the possibility that some form of musical long-term knowledge was developed before the exposure phase that we could not test using the behavioral data.

If we assume musical long-term knowledge was acquired in the first block of trials in Experiment 3, we need to also explain why there were no effects on the P3b-like component in the second block of trials since presumably participants would still possess this knowledge then. To this end, previous research^41^ showed that the amplitude of such a component can diminish after supervised training. The authors collected EEG data while participants listened to 10 repetitions of 16 excerpts from music pieces which either ended on a musically regular or musically irregular chord. The amplitude of the P3 component decreased during the learning trials.

The authors of that study^41^ attribute this decrease to the fact that participants became increasingly adept at predicting the irregular chord over the course of the learning trials. They liken this to the development of veridical knowledge about the stimuli that were used in the experiment rather than the knowledge about the irregularity of the chord. Similarly, participants in Experiment 3 may have gone through enough trials to have developed knowledge about which tones were expected as probe tones in the experiment.

Participants in our experiment may thus have evaluated how expected the probe tone was not in relation to the probe-tone context but in relation to all the other probe tones that they had already encountered. Meaning, the previously unexpected probe tone—unexpected because it was not part of the underlying music system—became expected because it had been encountered previously as a probe tone already. This decrease in “surprise” would then have led to a decrease of the ERP marker, resulting in its observed absence, despite the underlying knowledge that had already been gained about the music system itself.

In conclusion, our behavioral results indicate that listeners may develop musical long-term knowledge and our neurophysiological results imply that they do so at an incredibly fast pace, well within 160 short encounters with a new music system. It seems that not only do non-musicians possess sophisticated knowledge about the music around them but that they are also fast learners of unfamiliar music. Future research needs to benchmark shorter behavioral paradigms, with which musical long-term knowledge can be tested before neurophysiological correlates of the development of musical long-term knowledge can be investigated.

## Methods

All methods were carried out in accordance with relevant guidelines and regulations. All experiments were reviewed by the relevant ethics review board at Queen’s University, and data from each participant were collected only after their informed consent was obtained.

### Experiment 1

#### Participants

Thirty participants were recruited (23 female, 7 male, *M*_age_ = 21.4 years, *SD*_age_ = 2.81 years). The average years of music training (including school-based music instruction) was *M* = 6.07 years, *SD* = 4.08 years.

#### Procedure

Participants were fitted with a 128-channel Geodesic Sensor Net, a network of 128 electrodes connected within an elastic geodesic tension structure. Participants were asked to provide 80 probe-tone judgments. For each one of them, they heard a sequence of 34 tones followed by a single probe tone, and indicated on a two-alternative forced choice whether the probe tone fit or did not fit with the tone sequence heard before. This procedure is akin to the probe-tone paradigm used in music perceptual work to probe representations of the tonal hierarchy^6^. The tone sequences are described below. Stimuli were presented using E-Prime 2.0, running on a personal computer (Dell Optiplex 7020). During this task, EEG activity was recorded in reference to a vertex electrode with a sampling rate of 250 Hz. Impedances of each electrode were kept below 50 kΩ.

#### Stimuli

Tone sequences for this and the following experiments were constructed using the same sample recordings and time parameters. All tone sequences were generated in MATLAB using recordings of single tones on a Steinway & Sons grand piano B model from the online archive of the University of Iowa Electronic Music Studios^42^. Tones in tone sequences were all 150 ms long. Each tone sequence was immediately followed by an interstimulus interval of random length between 800 and 1200 ms, and then followed by a probe tone, which lasted 800 ms.

Eighty tone sequences each consisting of 34 tones were generated with the tones C4, D4, E4, F4, G4, and C5 occurring each with 14.7% probability, and the tones A4 and B4 occurring each with 6% probability to mimic a musical system with a hierarchical structure. Specifically, all tones are part of the C-major scale and can thus be interpreted as representative of a melody written in a 7-tone diatonic system. To prevent expectations about the length of each stimulus which could imply an underlying rhythmic structure, 25% of the tone sequences were shortened by cutting one tone, and 25% of the tone sequences were lengthened by adding a tone randomly drawn from the tones mentioned above. Four probe tones were used: C4 and G4, which were considered congruent with the tone distribution, and C♯4 and F♯4, which were considered incongruent with the tone distribution. C4 and G4 are diatonic tones; C♯4 and F♯4 are non-diatonic tones.

#### Data processing

Raw EEG signals were filtered with a 0.1–30 Hz bandpass digital filter to remove environmental noise, slow drifts, and high frequency muscle artifacts. Data were visually inspected to identify and remove bad channels. Time windows during recording with large or paroxysmal artifacts were also removed based on visual inspection. Independent components were then calculated using the EEGLAB toolbox^43^. Artifactual components due to movements or blinks were removed based on visual inspection of component activity and its correspondence to electrode activity across the recording period, component scalp maps, and the activity spectrum of the component. After removal of these artefactual independent components, data were recomposed using the remaining components. Previously removed channels were then interpolated based on the activity of neighboring channels, again using EEGLAB.

Epochs time-locked to the presentation of the probe tone were created from the processed data, such that they were 800 ms long, i.e., the length of the probe tone, with a 100 ms baseline. Epochs, in which activity exceeded 4 standard deviations at any electrode, were rejected to remove artifacts caused by drift or movement. After rejection, an average over all participants of 28.77 trials remained for each level of congruency (congruent: *M* = 28.77, *SD* = 3.58; incongruent: *M* = 28.77, *SD* = 3.47).

**Figure 7:**
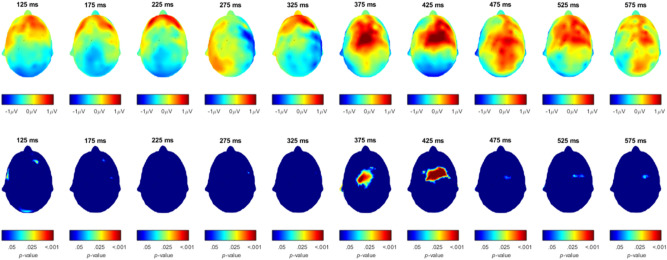
EEG activity following congruent probe tones subtracted from EEG activity following incongruent probe tones (upper row) and p-values of Wilcoxon signed rank tests of this difference (second row).

Figure 7 shows the raw difference between EEG activity following incongruent and congruent probe tones in the first row, and the *p*-value of Wilcoxon signed rank tests comparing this activity in the second row, such that *p*-values above *p* > 0.05 are shown in dark blue. Significant differences are indicated using a color scale which is shown underneath the plots.

Visual inspection of our data suggested possible effects of congruency at ROIs along the midline of the skull between 380 and 450 ms. ROIs were formed by averaging signals across electrodes around three midline electrodes, Fz, Cz, and Pz, based on the morphology of the EEG net (channel numbers indicate the electrode nomenclature of the Geodesic Sensor Net – frontal: 4, 5, 11 (Fz), 12, 16, 19; central: 7, 31, 55, 80 106, 129 (Cz); parietal: 61, 62 (Pz), 67, 72, 77, 78). Mean amplitude was calculated over 40 ms around the positive peak within the visually identified time window for each participant to account for potential individual differences in peak latency while guarding against distortions from high-frequency noise^44^. ANOVAs on mean amplitudes were calculated with location of the ROI as a factor (frontal, central, parietal) and Congruency as another factor (congruent, incongruent).

Probe-tone ratings for congruent and incongruent probe tones were formed by calculating the proportion of times that each participant regarded them as fitting. Probe-tone ratings were then compared using a paired samples *t*-test. To explore the relationship between behavioral and electrophysiological data, repeated measures correlations^45^ were calculated.

### Experiment 2

#### Participants

Thirty-four participants were recruited (25 female, 9 male, *M*_age_ = 22.44 years, *SD*_age_ = 5.59 years). The average number of years of music training was *M* = 5.32 years, *SD* = 4.39.

#### Procedure

Participants completed the same procedure as in Experiment 1. They also gave probe tone judgements in a separate block of 80 trials for non-diatonic tone sequences whose makeup will be detailed below. The order of the two blocks was counter-balanced among participants. EEG activity was recorded for both blocks.

#### Stimuli

The tone sequences for the additional block were constructed using the same sample recordings and time parameters described for Experiment 1. The tones used for the additional block of non-diatonic tone sequences were C4, D4, E4, F♯4, G♯4, and A♯4 each with 14.7% probability, and tones D♯4 and A4 each with 6% probability. The selection of tones assumed that whole-tone scales such as C–D–E–F♯–G♯–A♯ are rarely used in Western music. The same four probe tones C4, C4♯, F♯4, and G4 were used in this block though the congruency of two of the four probe tones was switched. While C4 and G4 are congruent with the previously used diatonic tone sequences, hereinafter referred to as a familiar music system, C4 and F♯4 were congruent with the unfamiliar music system. Conversely, C♯4 and F♯4 were considered incongruent with the familiar music system, whereas C4♯ and G4 were incongruent with the unfamiliar music system.

#### Data processing

EEG data was processed in the same manner as in Experiment 1. After processing, an average of 30.26 trials remained for each level of congruency at each level of distribution (familiar congruent: *M* = 30.58, *SD* = 3.45; familiar incongruent: *M* = 30.12, *SD* = 4.48; unfamiliar congruent: *M* = 29.96, *SD* = 2.94; unfamiliar incongruent: *M* = 30.4, *SD* = 2.77). The mean amplitude over 40 ms calculated around the positive peak in the time window defined through Experiment 1 (380 ms to 450 ms) was analyzed across the three midline ROIs (frontal, central, parietal).

Probe-tone ratings were formed by calculating the proportion of times a probe tone was judged as fitting. They were analyzed in an ANOVA with factors Tone Distribution (familiar, unfamiliar) and Probe Tone (C, C♯, F♯, G). To explore the relationship between behavioral and electrophysiological data, repeated measures correlations^44^ were calculated between responses separately for the familiar and unfamiliar block of tone sequences.

### Experiment 3

#### Participants

Forty participants were recruited (23 female, 17 male, *M*_age_ = 26.33 years, *SD*_age_ = 4.96 years). The average number of years of music training was *M* = 4.43 years, *SD* = 4.32.

#### Procedure

After providing consent, participants were fitted with a 128-chanel Geodesic Sensor Net as described above. Participants were then asked to provide 160 probe-tone judgments. In each, they heard a tone sequence followed by a single probe tone and indicated whether the tone fit or did not fit with the tone sequence heard before. Afterwards, participants were exposed to a continuous stream of tones for 30 min whose makeup will be described below as well. We refer to this phase as the exposure phase. Electrodes were checked for impedances at this point. Then participants gave another 160 probe-tone judgments. EEG activity was recorded during the probe tone judgment blocks.

#### Stimuli

Tone sequences were created in the same manner as the tone sequences of the unfamiliar music block in Experiment 2, i.e., with tones C4, D4, E4, F♯4, G♯4, and A♯4 occurring each with 14.7% probability (probe-tone category *C*), and tones D♯4 and A4 occurring each with 6% probability (probe-tone category *C*_P_). The continuous stream of tones participants heard between probe-tone judgment blocks, i.e., during the exposure phase, were formed with the tones C4, D4, E4, F♯4, G♯4, and A♯4 occurring each with 14.7% probability, and tones C♯4 and G4 each with 6% probability (probe-tone category *C*_E_). Tones F and B only occurred as probe tones (probe-tone category *C*~).

### Data processing

Data processing steps followed the pipeline outlined for Experiment 1. After rejection, an average of 30.19 trials remained for each probe-tone category in each probe-tone task block (before exposure: *C*: *M* = 30.16, *SD* = 3.67, *C*_P_: *M* = 29.97, *SD* = 3.84, *C*_E_: *M* = 29.68, *SD* = 3.93, *C*~: *M* = 29.82, *SD* = 3.70; after exposure: *C*: *M* = 31.16, *SD* = 3.76, *C*_P_: *M* = 30.21, *SD* = 3.84, *C*_E_: *M* = 30.32, *SD* = 3.90, *C*~: *M* = 30.18, *SD* = 4.21). The mean amplitude over 40 ms calculated around the positive peak in the time window defined through Experiment 1 (380 ms to 450 ms) was analyzed across the three midline ROIs (frontal, central, parietal) with additional factors Exposure denoting whether the probe tone was present during exposure or not (*C* and *C*_E_ vs *C*~ and *C*_P_), Probe-Tone Block denoting whether the probe tone was present during probe-tone judgment blocks or not (*C* and *C*_P_ vs *C*~ and *C*_E_), and Time denoting whether the probe-tone judgment was given before or after the exposure phase. We expected a significant interaction of Time, ROI, Exposure, and Probe-Tone Block such that the ERP marker would appear only after the exposure phase, that is to say, the mean amplitude would be greater for probe-tone category *C*~ than *C*_E_, at frontal and central electrodes.

To enable comparison to the data reported previously^31^ we analyzed the behavioral data in the same manner as it was analyzed in that paper, using a mixed-effects model, for all probe-tone judgments with the random effect of Participant, the fixed effects of Exposure, Probe-Tone Block, and Time. Here, we expected a significant three-way interaction between Time, Exposure, and Probe-Tone Block, such that ratings for probe-tone categories *C*~ and *C*_E_ would be significantly different only after the exposure phase.

Repeated measures correlations were again calculated between behavioral and electrophysiological data separately for the block of trials before and the block of trials after the exposure phase, akin to the analysis described for Experiment 2.

## Data Availability

The datasets generated and analyzed during the current study are available from the corresponding author by request.
